# Temporal Trends in Phenotypic Macrolide and Nonmacrolide Resistance for *Streptococcus pneumoniae* Nasopharyngeal Samples Up to 36 Months after Mass Azithromycin Administration in a Cluster-Randomized Trial in Niger

**DOI:** 10.4269/ajtmh.23-0431

**Published:** 2023-10-02

**Authors:** Ashley Hazel, Ahmed M. Arzika, Amza Abdou, Elodie Lebas, Travis C. Porco, Ramatou Maliki, Thuy Doan, Thomas M. Lietman, Jeremy D. Keenan, Seth Blumberg

**Affiliations:** ^1^ F. I. Proctor Foundation, University of California, San Francisco, California;; ^2^ The Carter Center, Niamey, Niger;; ^3^ Programme Nationale de Santé Oculaire, Niamey, Niger;; ^4^ School of Medicine, University of California, San Francisco, California

## Abstract

Azithromycin mass drug administration decreases child mortality but also selects for antibiotic resistance. Herein, we evaluate macrolide resistance of nasopharyngeal *Streptococcus pneumoniae* after azithromycin MDA. In a cluster-randomized trial, children 1–59 months received azithromycin or placebo biannually. Fifteen villages from each arm were randomly selected for antimicrobial resistance testing, and 10–15 randomly selected swabs from enrolled children at each village were processed for *S. pneumoniae* isolation and resistance testing. The primary prespecified outcome was macrolide resistance fraction for azithromycin versus placebo villages at 36 months. Secondary non-prespecified outcomes were comparisons of azithromycin and placebo for: 1) macrolide resistance at 12, 24, and 36 months; 2) nonmacrolide resistance at 36 months; and 3) suspected-*erm* mutation. At 36 months, 423 swabs were obtained and 322 grew *S. pneumoniae*, (azithromycin: 146/202, placebo: 176/221). Mean resistance prevalence was non-significantly higher in treatment than placebo (mixed-effects model: 14.6% vs. 8.9%; OR = 2.0, 95% CI: 0.99–3.97). However, when all time points were evaluated, macrolide resistance prevalence was significantly higher in the azithromycin group (β = 0.102, 95% CI: 0.04–0.167). For all nonmacrolides, resistance prevalence at 36 months was not different between the two groups. Azithromycin and placebo were not different for suspected-*erm* mutation prevalence. Macrolide resistance was higher in the azithromycin group over all time points, but not at 36 months. Although this suggests resistance may not continue to increase after biannual MDA, more studies are needed to clarify when MDA can safely decrease mortality and morbidity in lower- and middle-income countries.

## INTRODUCTION

Mass azithromycin administration has been shown to reduce mortality by up to 25% for preschool children in several African communities^1^^,^^2^ and reduces population burden of endemic infections like trachoma.^3^^,^^4^ Mass drug administration (MDA) continues to be an effective intervention for underserved and poorly resourced communities,^5^^,^^6^ but MDA can also select for community-wide prevalence of antimicrobial resistance. The long-term risk of antibiotic resistance is unclear and may be affected by duration, frequency, and coverage of MDA protocols.^7^^,^^8^

The cluster-randomized MORDOR trial provides an opportunity to quantify the emergence of antibiotic resistance after villages in Niger were cluster randomized to receive either twice-yearly azithromycin or placebo for children aged 1 to 59 months for 2 years. Macrolide and nonmacrolide resistance in nasopharyngeal *Streptococcus pneumoniae* as well as resistance determinants in the gut were monitored at 0, 12, 24, and 36 months of trial initiation. Phenotypic macrolide resistance of nasopharyngeal *S. pneumoniae* was 4 times higher at 24 months in the villages that received azithromycin treatment (azithromycin villages) than in the villages that received placebo (placebo villages).^9^ Additionally, genetic determinants of macrolide resistance were higher in the gut microbiota of children in azithromycin villages at 36 months than of children in placebo villages; however, the prevalence of genetic determinants of macrolide resistance in the gut did not increase from 36 months to 48 months.^10^ The presence of genetic determinants of resistance might not reflect active phenotype and may demonstrate different longitudinal trends.^11^ Thus, although the trend in genetic resistance suggests a long-term higher risk of phenotypic resistance in communities that receive MDA, the peak level of phenotypic risk after MDA and whether resistance persists over time are unclear.

To characterize the temporal dynamics and persistence of phenotypic resistance, we examined macrolide and nonmacrolide resistance from the MORDOR trial in children’s nasopharyngeal samples from azithromycin and placebo villages 36 months after trial initiation. Our primary intention was to determine whether resistance rates remained higher at 36 months in azithromycin villages than in placebo villages and whether resistance rates continued to increase beyond the 12- and 24-month prevalence values reported by Doan et al.^9^

## MATERIALS AND METHODS

### Trial design.

The MORDOR (Macrolides Oraux pour Réduire les Décès avec un Oeil sur la Résistance) trial is a cluster-randomized trial administered in 3 African countries—Niger, Malawi, and Tanzania. Randomization occurred at the village level, and mortality rates for children aged 1 to 59 months were evaluated according to whether the village’s children received azithromycin or placebo every 6 months for 4 years (8 doses overall). Across all study countries, 1,533 villages were randomized into azithromycin or placebo arms, and 190,238 children were enrolled.^1^

**Figure 1.**
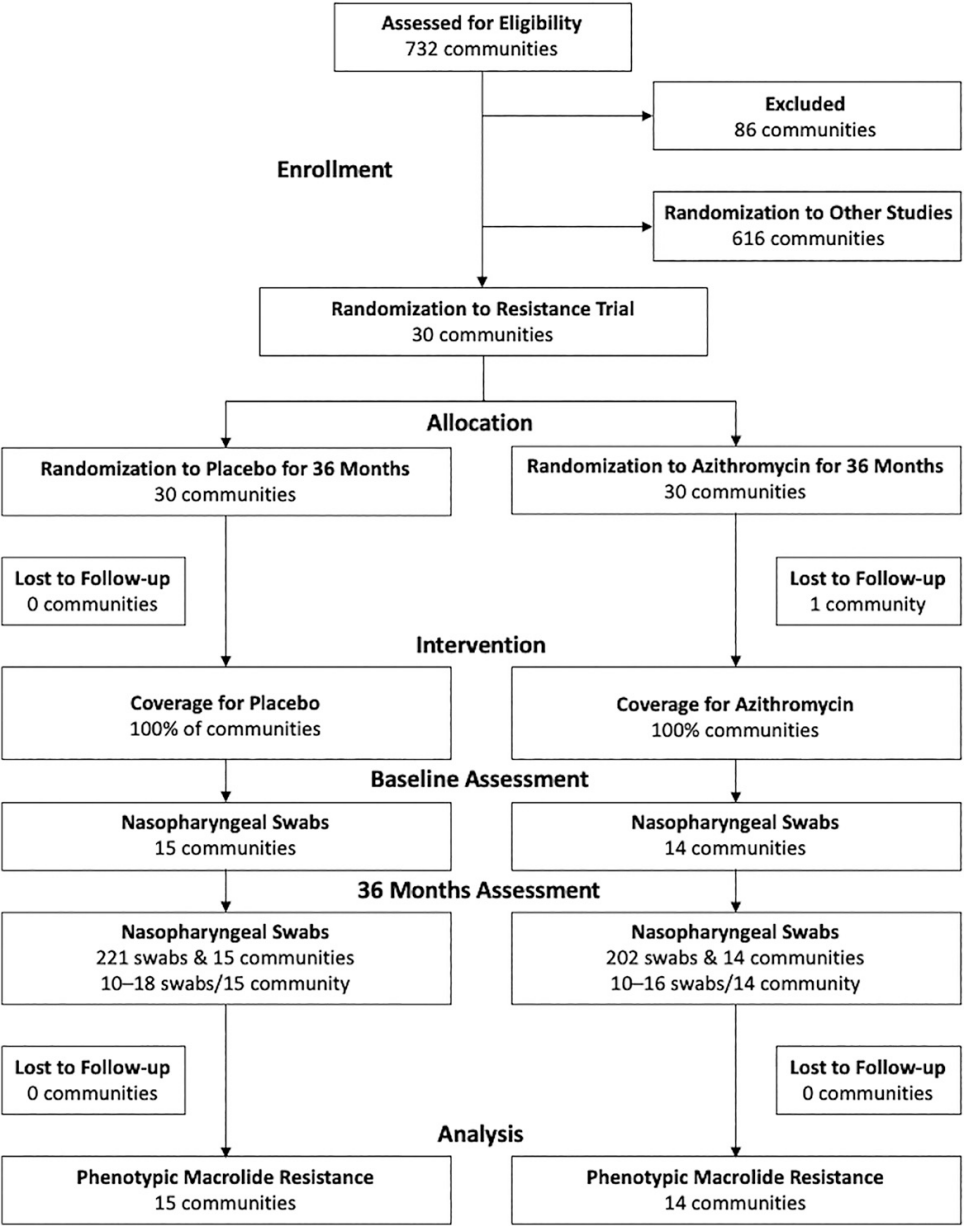
Community and participant flow for trial intervention and antimicrobial resistance assessment at 36 months.

In Niger, 15 villages from each study arm (30 total villages) were randomly assigned to be additionally evaluated for resistance morbidity, and resistance was evaluated posttreatment initiation at three time points: 12, 24, and 36 months. The complete MORDOR protocol and CONSORT metrics are available in the Supplemental Materials. Approximately 15 swabs per village at 36 months were analyzed, and approximately 10 swabs per village were analyzed at 12 and 24 months (Table 1 and Figure 1). Cluster number and cluster size determinations are described in detail in the trial protocol (see Supplemental Materials). Our target sample size assumed 12% baseline resistance and 80% power to detect an 18% (range, 12% to 30%) difference from baseline. See the MORDOR Statistical Analysis Plan in Supplemental Materials for more details.

**Table 1:** Numbers of nasopharyngeal swabs and *S. pneumoniae* isolates at each time point for azithromycin and placebo clusters

Time point	Azithromycin		Placebo	
	Swabs	Isolates grown (%)	Swabs collected	Isolates grown (%)
0 months	150	18 (12.0)	150	10 (6.7)
12 months	144	84 (58.3)	150	82 (54.7)
24 months	150	81 (54)	150	82 (54.7)
36 months	202	147 (72.8)	221	176 (79.6)

### Trial oversight.

The MORDOR trial and morbidity study received ethical approval from the Institutional Review Board of the University of California, San Francisco, the Committee for Human Research, and the Niger Ministry of Health. All trial activities complied with the principles of the Declaration of Helsinki. Guardians provided oral informed consent for children’s participation, in lieu of written consent, because of low literacy rates in the study area.

### Settings and participants.

Trial intervention and swab collection were undertaken in Niger from December 2014 through June 2019, in the Loga and Boboye divisions. Villages with population sizes between 200 and 2,000 residents were eligible for trial inclusion. Children aged 1 to 59 months who weighed ≥ 3,800 g were eligible to receive azithromycin or placebo.

### Randomization.

Randomization to azithromycin or placebo occurred at the village level, with randomization sequences generated with R software (version 3.5.1; R Foundation for Statistical Computing). Village assignment to azithromycin or placebo was masked to all trial staff (coordinators, field workers, and investigators), except for the trial biostatistician who performed randomization.

### Interventions.

The MORDOR interventions involved twice yearly administration of either azithromycin or placebo for 4 years, under observation by trained study personnel. Oral suspension of azithromycin (20 mg/kg) or placebo was administered every 6 months, starting at baseline (0 months). Children with known macrolide allergies were excluded from the trial.

### Sample collection.

Nasopharyngeal swabs were obtained annually from 40 randomly selected children in each village. Sampled children were independently selected year to year. At baseline, 12 months, and 24 months, 10 swabs per village were randomly selected for culture via broth dilution assay. At 36 months, approximately 15 swabs (mean, 14.6; range, 10 to 15) were randomly selected for culture.

### Outcomes.

Our primary prespecified outcome was the fraction of phenotypic macrolide-resistant *Streptococcus pneumoniae* from isolates from children aged 1 to 59 months in azithromycin versus placebo arms at 36 months. Our additional non-prespecified outcomes were to compare azithromycin and placebo groups for 1) the fraction of nonmacrolide-resistant *S. pneumoniae* in azithromycin versus placebo arms at 36 months, 2) the fraction of erythromycin-resistant *S. pneumoniae* in azithromycin versus placebo arms for all combined time points (12, 24, and 36 months), and 3) the difference between azithromycin and placebo groups for percentage of suspected *erm* and suspected *mef* mutations among *S. pneumoniae* isolates at 36 months.

### Statistical analyses.

To compare macrolide resistance in the treatment- and placebo-controlled arms, we tested for evidence of a significant difference in erythromycin resistance prevalence by using a permutation test for *P* value estimation. We also fit logistic models of individual-level results for resistant *S. pneumonia* (binary outcome variable: resistant = 1, sensitive = 0) with village as a random effect as sensitivity analyses. We used the Benjamini and Hochberg method^12^ to estimate the *P* value after adjustment for repeat measures. For all analyses, we used the total number of *S. pneumoniae* isolates grown in each village as our denominator to determine mean proportion of resistance, and all statistical comparisons between clustered study arms were based on the average of the proportions for each village (as represented in Supplemental Table 1). To test for temporal trends in erythromycin resistance between azithromycin and placebo groups, we fit linear mixed-effects models with fixed effects for time and treatment arm and random effect for village.

Using the same statistical techniques described above, we estimated prevalence differences for other common antimicrobials—clindamycin, penicillin, doxycycline, and trimethoprim-sulfamethaxazole.

We also assessed whether resistance patterns consistent with suspected *erm* mutation (assessed as phenotypically erythromycin resistant and clindamycin resistant) or *mef* mutation (erythromycin resistant and clindamycin susceptible) were different between the azithromycin and placebo groups. We fit two logistic regressions with random effects for each village. First, we compared *erm* mutation*-*suspected status to non-*erm* mutation-suspected status (i.e., clindamycin susceptible) between azithromycin and placebo groups. Second, we compared *mef*-suspected status to erythromycin- and clindamycin-susceptible status between the two arms, because among the erythromycin-resistant strains alone, we cannot differentiate between *mef* and *erm* mutation-suspected resistance determinants. All analyses were conducted in R 4.2.2.^13^

## RESULTS

### Participant flow and recruitment.

For the 4-year (8-dose) intervention, mean coverage for azithromycin and placebo was 83.2% ± 16.4% and 86.6% ± 12.0%, respectively. One village in the azithromycin arm withdrew from the trial after the 24-month time point (final village sample *N* = 29).

A total of 202 nasopharyngeal swabs in the azithromycin treatment group and 221 swabs in the placebo group were cultured for the 36-month time point (details for swab collection for 12 and 24 months are reported in reference 9). Participant recruitment and loss to follow-up data are presented in Figure 1.

### Outcomes and estimation.

At 36 months, *S. pneumoniae* growth was marginally greater in placebo (147/202, 72.8%) than azithromycin (176/221, 79.6%, *P* = 0.07; Supplemental Table 1). Growth rates did not differ between azithromycin and placebo arms in other years; however, the percentage of isolates grown at 36 months was higher than that at 12 and 24 months (Table 1). A cold-chain issue with sample transport resulted in unrealistically low isolate growth at baseline. Of 300 total samples tested, only 28 samples grew *S. pneumoniae* (18 in azithromycin arm, 10 in placebo arm); thus, we did not include baseline values in our analyses (Table 1).

Macrolide resistance was 14.6% (95% CI, 8.5–21.3) in the *S. pneumoniae* samples from the azithromycin group and 8.9% (95% CI, 4.8–13.2) (Table 2) in the placebo group, but the difference in mean prevalence at 36 months was not significant (random effects model: odds ratio [OR] = 2.0 [0.99–3.97]; repeated-measures adjusted *P* = 0.19) (Table 3). Similarly, we did not find any significant differences in nonmacrolide resistance between azithromycin and placebo arms at 36 months in a repeated-measures analysis (Tables 2 and 3).

**Table 2:** Proportion of nasopharyngeal pneumococcal antibiotic resistance at 36 months

Antibiotic class	Azithromycin	Placebo
	% (95% CI*)	
Erythromycin	14.6 (8.5–21.3)	8.9 (4.8–13.2)
Clindamycin	7.8 (3.5–12.5)	4.2 (1.2–8.1)
Penicillin	34.6 (24.1–46.6)	22.0 (16.0–28.0)
Trimethoprim-sulfa- methaxazole	90.3 (84.7–95.2)	84.5 (80.1–89)
Doxycycline	66.0 (55.8–76.5)	52.0 (45.5–58.5)
Linezolid	0.0 (0–0)	0.0 (0–0)
Ceftriaxone	1.0 (0–2.1)	0.0 (0–0)
Vancomycin	0.0 (0–0)	0.0 (0–0)
Levofloxacin	0.0 (0–0)	0.0 (0–0)
Meropenem	0.0 (0–0)	0.0 (0–0)

* 95% CIs calculated via bootstrap method.

**Table 3:** Permutation test and logistic regression of resistance proportion between azithromycin and placebo arms at 36 months

Antibiotic class	Permutation test *P* value	Adjusted *P* value*	Logistic models (OR [95% CI])†
Erythromycin	0.15	0.19	2.0 (0.99–3.97)
Clindamycin	0.26	0.26	2.0 (0.67–7.05)
Penicillin (oral)	0.07	0.18	1.6 (0.90–3.12)
Trimethoprim-sulfa- methaxazole	0.13	0.19	1.8 (0.93–3.81)
Doxycycline	0.04	0.18	1.6 (0.97–2.67)
Linezolid	–	–	–
Ceftriaxone	–	–	–
Vancomycin	–	–	–
Levofloxacin	–	–	–
Meropenam	–	–	–

OR = odds ratio

* Adjusted for multiple comparisons using the Benjamini and Hochberg false discovery rate method.

† Binomial outcome [erythromycin resistant: yes (1) or no (0)] for all isolates with random effect for village.

**Figure 2.**
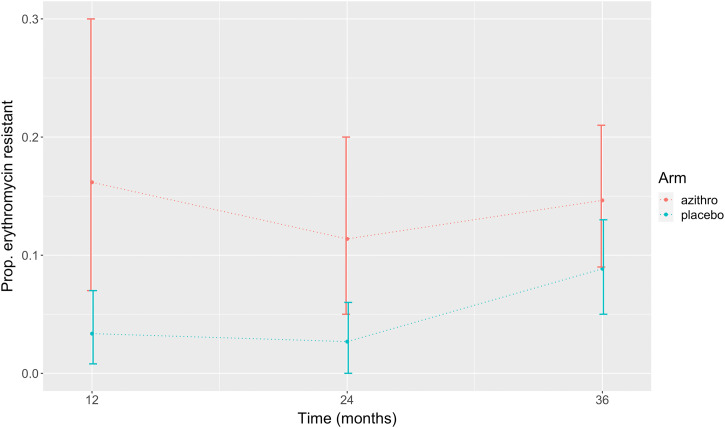
Proportion of erythromycin-resistant *Streptococcus pneumoniae* isolates in treatment versus placebo arms at three assessment time points (12, 24, and 36 months). Baseline values were not included in statistical analyses because of poor isolate growth.

A linear mixed-effects model was used to evaluate erythromycin resistance across all time points (12, 24, and 36 months). We found significant evidence of higher prevalence in the azithromycin group than in the placebo group (fixed effect of arm, 0.102; 95% CI, 0.04–0.17), but we did not observe an interaction effect between study arm and time (fixed effect of interaction, 0.606; 95% CI: −0.01 to 0.00) (Figure 2).

The proportion of suspected *erm* mutations among *S. pneumoniae* isolates was higher in azithromycin villages (7.8%) than placebo villages (4.2%) but not significantly (OR = 1.87; 95% CI, 0.55–7.25). We also found evidence of a higher proportion of suspected *mef* mutations in the azithromycin groups than the placebo groups, although significance was not reached (OR = 2.06; 95% CI, 0.71–6.01) (Table 4).

**Table 4:** Suspected *erm* mutation prevalence in study arms at 36 months

Resistance profile	Number of mutations in azithromycin arm (% phenotype)	Number of mutations in placebo arm (% phenotype)
Erythromycin and clindamycin	11 (58)	8 (42)
Erythromycin only	12 (60)	8 (40)
Clindamycin only*	1 (100)	0 (0)
None	123 (42)	169 (58)
	147	185

* Removed from regression models.

## DISCUSSION

Phenotypic resistance prevalence values for macrolide and nonmacrolide antibiotics in *S. pneumonia* were increased 36 months after initiation of mass azithromycin administration in treatment villages compared with those in placebo villages, but the difference did not meet statistical significance. However, a comparison between study arms that included all time points indicated that prevalence of erythromycin resistance was significantly higher in azithromycin treatment villages. Erythromycin resistance prevalence increases marginally over the evaluation time points and may be saturating at approximately 15% in azithromycin villages. This “flattening out” pattern at 36 months is different from the rapid increase in resistance observed after azithromycin administration for trachoma treatment in other studies, where azithromycin is administered more frequently and to a broader scope of the population.^14^^,^^15^ Logistic-growth estimates^16^ based on 2-year trends from the MORDOR trial^1^^,^^2^ indicate that resistance after 5 years of MDA for trachoma could reach 80%.^8^ However, longer posttrial follow-up also showed decreasing macrolide resistance prevalence.^17^^,^^18^ Our 36-month data from the MORDOR trial indicate that resistance also decreases over time but that it may peak at a lower prevalence than after MDA with greater frequency of intervention. We cannot disentangle potential spillover effects that could explain increases in resistance prevalence in placebo villages from decreasing selection pressure that could explain plateaued prevalence in azithromycin villages. Further studies and longer follow-up are needed to understand the long-term effects of different MDA interventions both with ongoing MDA and after MDA cessation.

Our results show some interesting consistencies and differences with resistance determinants from gut microbiota sampled from the same individuals at 36 months. Resistance determinants of macrolides and nonmacrolides in gut microbiota were significantly higher in the azithromycin treatment group than in the placebo group,^10^ whereas we found no significant differences in phenotypic resistance in *S. pneumococcus* obtained from nasopharyngeal samples. Direct comparisons between phenotype and genotype require caution because genetic resistance determinants are a composite representative of all gut bacteria,^9^^,^^10^ whereas phenotype was only measured for *S. pneumoniae.* Especially since resistance determinants in gut microbes could impact clinically important pathogens such as *Escherichia coli* and *Salmonella*, the comparison of clinical applicability and economic practicality of genetic versus phenotypic surveillance for resistance deserves further attention.

It is not clear why resistance increased in the placebo villages. One possibility is that children gained exposure to resistant strains at schools where children from placebo and azithromycin treatment villages mix. Another possibility is that children in placebo villages experienced greater antibiotic exposure after the trial due to a general global trend of increased antibiotic use. In both explanations, social mixing among children in different intervention clusters would lead to greater exposure. A better understanding of the geographic proximities, seasonal mobility (e.g., school, work), and other measures of social connectivity among communities would help future efforts to predict resistance emergence and spread.^19^ Data on overall use of antibiotics in the community and trend over time would also be useful.

A major limitation of our study, because this trial was randomized at the village level and children were selected at random each year to provide a nasopharyngeal swab, is that we can only make inferences about prevalence at the community level. Longitudinal studies on an individual level could yield additional information, such as how often individuals with resistant phenotypes revert to susceptible and the timescale of such transitions.

This study provides perspective on the risk-benefit of MDA distribution programs. Although the study lacks the power to make definitive statistical statements regarding the relationship between MDA and antibiotic emergence, a few qualitative conclusions are supported. Namely, MDA does appear to promote antibiotic resistance, but the population-level impact appears to plateau. To concretely evaluate the short-term benefits of MDA programs versus the long-term risks of antibiotic resistance, more studies are needed to evaluate the clinical significance of increased community-level colonization with resistant organisms and to determine how long the increased prevalence of antibiotic resistance persists after MDA cessation. It is also important to further evaluate the extent to which MDA distribution in a particular community may impact the risk of antibiotic infections in neighboring communities. These types of studies will help clarify when MDA programs can be safely deployed to save lives and decrease morbidity in lower- and middle-income countries.

## Supplemental Materials


Supplemental materials
